# Genetic diversity of *Echinococcus vogeli* in the western Brazilian Amazon

**DOI:** 10.1590/0074-02760190149

**Published:** 2019-09-26

**Authors:** Daniel Daipert-Garcia, Márcio Galvão Pavan, Leandro Batista das Neves, Fernanda Barbosa de Almeida, Nilton Ghiotti Siqueira, Guilherme Brzoskowski dos Santos, Tuan Pedro Dias-Correia, Henrique Bunselmeyer Ferreira, Rosângela Rodrigues-Silva

**Affiliations:** 1 Fundação Oswaldo Cruz-Fiocruz, Instituto Oswaldo Cruz, Laboratório de Helmintos Parasitos de Vertebrados, Rio de Janeiro, RJ, Brasil; 2 Fundação Oswaldo Cruz-Fiocruz, Instituto Oswaldo Cruz, Laboratório de Mosquitos Transmissores de Hematozoários, Rio de Janeiro, RJ, Brasil; 3 Fundacão Hospital Estadual do Acre, Rio Branco, AC, Brasil; 4 Universidade Federal do Acre, Rio Branco, AC, Brasil; 5 Universidade Federal do Rio Grande do Sul, Centro de Biotecnologia, Laboratório de Biologia Molecular de Cestódeos, Porto Alegre, RS, Brasil; 6 Universidade Federal do Rio Grande do Sul, Centro de Biotecnologia, Laboratório de Genômica Estrutural e Funcional, Porto Alegre, RS, Brasil

**Keywords:** Echinococcus vogeli, polycystic echinococcosis, genetic diversity, cytochrome oxidase I, population genetics

## Abstract

Human polycystic echinococcosis is a parasitic infection caused by the larval stage of *Echinococcus vogeli*, which occurs in rural areas of Central and South America. Until now, little information on the genetic variability of *E. vogeli* is available. Here, 32 samples from human-excised *E. vogeli* cysts had a 396-bp sequence of the mitochondrial cytochrome oxidase I (COI) gene sequenced and compared to another 17 COI sequences representing nine *Echinococcus* species. A Bayesian COI tree revealed that all *E. vogeli* sequences formed a monophyletic and well-supported clade with an *E. vogeli* reference sequence. The occurrence of geographically restricted *E. vogeli* COI haplotypes suggests retention of ancestral polymorphisms with little migration in Acre, Brazil.

In Brazil, despite significant improvements in sanitary conditions observed in the last decade, there are still several parasitic diseases with high prevalence, especially in less developed areas.^1^ ^)^ Echinococcosis is a parasitic disease included in the list of neglected tropical diseases by the World Health Organization,^2^ and two forms of this anthropozoonosis are prevalent in Brazil, cystic echinococcosis (CE) in the South,^3^ and polycystic echinococcosis (PE) in the North.^4^ The different forms of echinococcosis are caused by the larval stages (metacestodes) of flatworms belonging to the genus *Echinococcus* Rudolphi, 1801 (Cestoda; Taeniidae), which infect humans and other mammals. They grow as one or multiple cysts or vesicles located most often in the liver and lungs of intermediate hosts.^5^

*Echinococcus vogeli* is the etiological agent of PE, which is endemic in the Neotropical region, including the North region of Brazil, and has a significant impact in terms of morbidity and mortality in the affected human populations.^6^ Medium-sized rodents are the intermediate hosts, and dogs (wild or domestic) are the definitive hosts of *E. vogeli*.^7^ The total number of human cases of PE reported (220 until 2015) is probably only a small fraction of the current infections, since several countries where the disease occurs do not have cumulative reporting and there is still considerable difficulty in the diagnosis and treatment of the disease, primarily in remote places.^8^ ^,^ ^9^

Studies on molecular genetics and evolutionary ecology combined with traditional taxonomy based on morphological characters have an essential role in understanding the biodiversity of the *Echinococcus* genus and the differentiation of species. It is estimated that the ancestral node of the genus *Echinococcus* dates from ~6 million years ago. Previous reports on the genus *Echinococcus* evolutionary history showed that Neotropical *E. vogeli* and *Echinococcus oligarthra* (the etiological agent of unicystic echinococcosis) were the first to diverge within the clade. They reached South America after the formation of the Isthmus of Panama, along with the immigration of wild canids and felids from North America.^10^

It has been recognised that intraspecific genetic variations can influence several factors in parasites, such as life cycle patterns, host specificity, development time, transmission dynamics, sensitivity to chemotherapeutics, antigenicity, and disease-causing ability.^5^ The elucidation of intraspecific genetic variation has greatly contributed to the characterisation of local populations and has allowed a better understanding of the parasite-host relationship and clinical manifestations of the disease, serving as the basis for the identification of important antigens and the development of immunodiagnostic assays and vaccines.^11^

Intraspecific or strain variations are considered a common feature of the genus *Echinococcus* and have been mainly characterised in *Echinococcus granulosus* sensu lato (the etiological agent of CE) and *Echinococcus multilocularis* (the etiological agent of alveolar echinococcosis).^10^ Several mitochondrial genetic variants differing in epidemiologically relevant characters have been described in these species.^12^ ^)^ However, for the Neotropical *Echinococcus* species (*E. vogeli* and *E. oligarthra*), little is known regarding intraspecific genetic variation. Therefore, to provide information on possible genetic variation in *E. vogeli*, we surveyed the genetic variability of metacestodes from PE patients. The survey was based on a 396-bp mitochondrial DNA sequence from the cytochrome-C-oxidase subunit 1 gene (COI), widely used in molecular studies of the genus *Echinococcus*.^13^

Thirty-two human isolates of surgically excised *E. vogeli* cysts [Supplementary data (Table)] were obtained from PE patients located in eight municipalities of the state of Acre, Brazil [Supplementary data (Figure, Table)]. The surgical procedure was part of the treatment and all samples (cysts) were collected with the patients’ agreement, according to the ethical standards of the Instituto Oswaldo Cruz, Fundação Oswaldo Cruz, Rio de Janeiro, RJ, Brazil and to the Helsinki Declaration of 1975 (revised in 2008).

Cysts were provisionally stored at -20ºC just after the surgical procedure and defrosted for molecular analysis. For each sample, total DNA was extracted from germinal membranes using the QIAamp DNA Mini Kit (QIAGEN, Hilden, Germany), following the manufacturer’s instructions. A 396-bp sequence of the COI sequence gene was amplified by polymerase chain reaction (PCR) from each *E. vogeli* isolate DNA sample. PCR was performed according to Bowles et al.,^14^ with modifications in reagent concentrations according to Sánchez et al.^15^ and cycling conditions as described in Santos et al.^16^ The amplicons were purified using the illustra™GFX™ PCR DNA kit (GE Healthcare, Little Chalfont, United Kingdom), following the manufacturer’s instructions. Both DNA strands were sequenced using the same PCR primers and the PrimTM ABI BigDye Terminator Cycle sequencing kit (Applied Biosystems, Foster City, USA), according to the manufacturers protocol. Sanger sequencing of amplicons was performed with an automated DNA sequencer ABI 3730 analyser (Applied Biosystems, Foster City, USA). Primer sequences were removed and a consensus sequence from the forward and reverse strands was assigned with SeqMan v. 7.1 (DNASTAR, Madison, USA).

Forty-nine 396-bp COI sequences (32 *E. vogeli* sequences from this study, and 17COI sequences from GenBank representing nine *Echinococcus* species (Figure) were included in the Bayesian phylogenetic tree reconstruction under the coalescent model inferred in BEAST v. 1.8.^17^ Three independent runs were performed for 5 × 10^7^ generations, sampling every 50,000 generations. Convergence of parameters and proper mixing were confirmed through the calculation of effective sample sizes (ESS) in Tracer v. 1.6;^18^ ESS estimates above 10^4^ were considered reliable.^19^ The best-fit model of nucleotide substitution was determined with jModel test v. 2.^20^ Molecular diversity indices of the number of segregating sites (S), the number of haplotypes (N_H_), haplotype diversity (H_D_), and nucleotide diversity (π) were computed in Dna SP v. 5^21^ for each sampling site, as well as deviations from neutrality, with Fu’s Fs^22^ and Tajima’s D^23^ tests. Sequence divergence between populations was calculated in Mega-X.^24^ A median-joining network^25^ was constructed with Network v. 4.6 (Fluxus Technology Ltd. 2008) for a better visualisation of the relationships between COI haplotypes.

**Figure:**
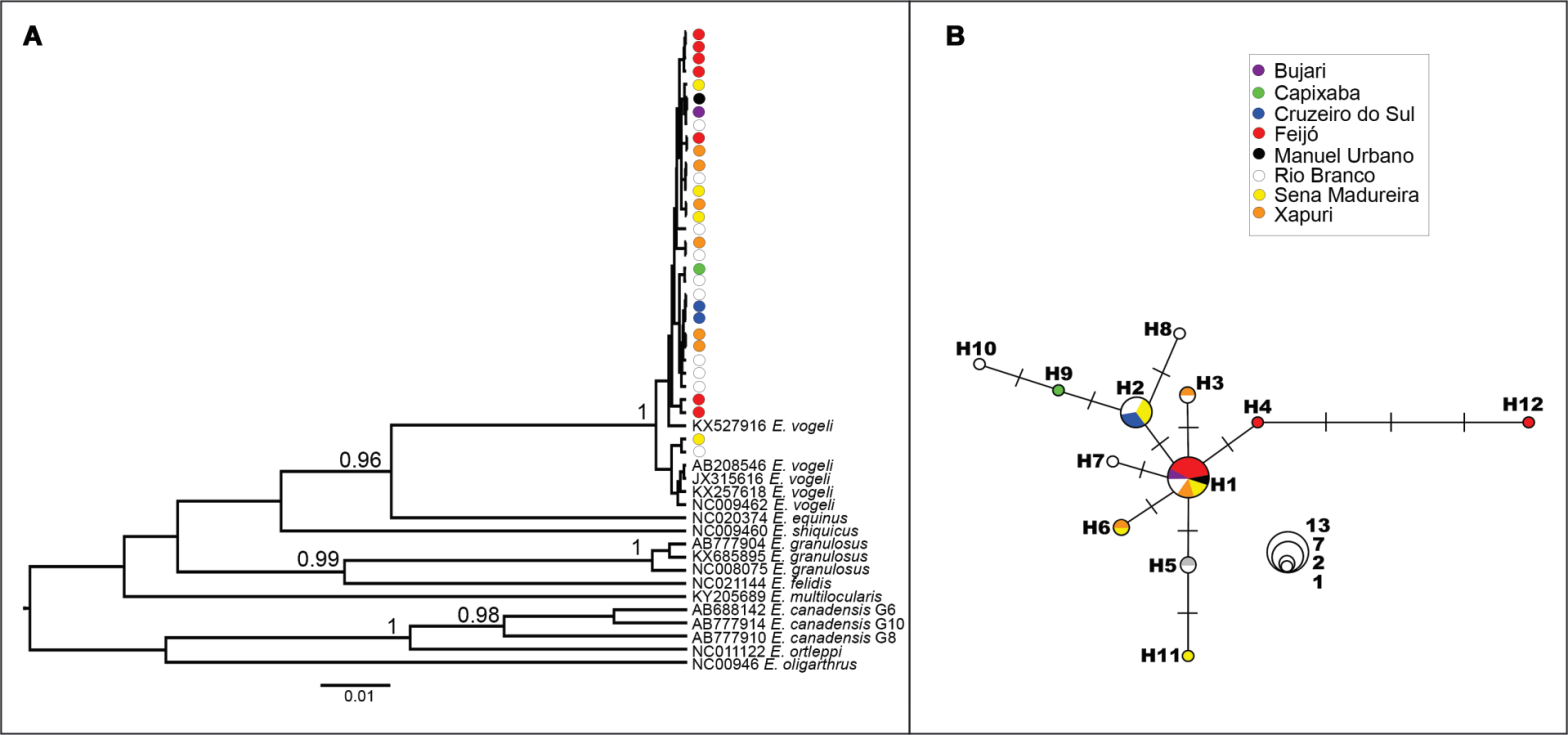
Bayesian maximum clade credibility tree reconstructed using a396-bp cytochrome oxidase I (COI) sequence of 49 *Echinococcus* specimens. GenBank accession numbers of *Echinococcus vogeli* sequences generated in this study: MK791154 to MK791185. The accession numbers of sequences retrieved from GenBank are shown in branch tips. Posterior probabilities above 0.9 are shown for key nodes. (B) Haplotype network based on COI sequences. Circle sizes are proportional to haplotype frequency. Each dash represents a mutational step.

The Tamura and Nei model with four gamma categories (TrN+Γ) was selected as the best evolutionary model for the data, following the Akaike and Bayesian information criteria. The Bayesian COI tree (Figure A) revealed that all *Echinococcus* sample sequences generated in this study formed a monophyletic and well-supported clade with an *E. vogeli* reference sequence (KX527916), further corroborating the species identity of the collected samples. This phylogenetic reconstruction disclosed short branches (i.e.low sequence divergence) among *E. vogeli* samples. Only two samples from Sena Madureira and Rio Branco clustered in a separate clade (PP = 1.0), along with another four *E. vogeli* sequences retrieved from GenBank (AB208546, JX315616, KX257618 and NC009462).

The molecular divergence of *E. vogeli* samples varied between zero and 0.8%, as expected for intraspecific comparisons. The most divergent sequence was from Capixaba (0.25-0.69%) and the least divergent sequence was from Bujari (0.10-0.27%). Inspection of the sequences revealed 12 polymorphic sites and 12 haplotypes, with five haplotypes (H1-H3, H5 and H6) shared between different localities (Figure B). Molecular diversity indices (Table) showed high haplotype diversity in three localities (H_D_ = 0.833-0.911) and nucleotide diversity (H_D_ = 0.0025-0.0044) comparable to *E. vogeli* COI data obtained in a previous study (H_D_ = 0.0027-0.0044).^16^

**TABLE:** Cytochrome oxidase I (COI)-based molecular diversity indices for the *Echinococcus vogeli* samples collected in eight localities of the state of Acre, Brazil

Locality	Geographical coordinates	N	S	N_H_	H_D_	π	Haplotypes
Bujari	67° 57’ 08’’W, 9° 49’ 50”S	1	0	1	0	0	H1
Capixaba	67° 40′ 33″W, 10° 34′ 22″ S	1	0	1	0	0	H9
Cruzeiro do Sul	72° 40′ 12″ W, 7° 37′ 51″ S	2	0	1	0	0	H2
Feijó	70° 21′ 14″ W, 8° 9′ 50″ S	7	4	3	0.524 (± 0.21)	0.0034 (± 0.002)	H1, H4, H12
Manuel Urbano	69° 15′ 36″ W, 8° 50′ 20″ S	1	0	1	0	0	H1
Rio Branco	67° 48’ 30’’W, 6° 58’ 32”S	10	7	7	0.911 (± 0.08)	0.0044 (± 0.009)	H1, H2, H3, H5, H7, H8, H10
Sena Madureira	68° 39’ 28’’W, 09° 04 ’02”S	6	4	4	0.867 (± 0.13)	0.0039 (± 0.001)	H1, H2, H6, H11
Xapuri	68° 30’ 16’’W, 10° 39’ 06”S	4	2	3	0.833 (± 0.22)	0.0025 (± 0.009)	H1, H3, H6
*E. vogeli* (this study)		32	12	12	0.796 (± 0.06)	0.0037 (± 0.001)	

N: sample size; S: number of segregating sites; N_H_: number of haplotypes; H_D_: haplotype diversity; π: nucleotide diversity.

Haplotypes derived from the COI sequences disclosed a network (Figure B) with weak geographic structure, since localities that are close and far apart (50-650km) shared the same haplotypes. The network had two central haplotypes (H1 and H2) that were very abundant and widespread, and to which seven other less common haplotypes were closely related (1 mutational step). The most frequent haplotype (H1, N = 13) was shared with specimens from all localities, except for Capixaba. The other common haplotype (H2, N = 7) was shared with specimens from Cruzeiro do Sul, Rio Branco and Sena Madureira. These geographically restricted haplotypes suggest sudden population expansion or retention of ancestral polymorphism with little migration. Since neutrality tests did not indicate significant departures from neutrality (p > 0.05), it is highly probable that the weak geographic structure of the network reflects retention of ancestral polymorphisms and restricted gene flow in Acre, Brazil.

High genetic variability and overall low levels of genetic structure have been shown for species of the *Echinococcus* genus. For instance, Sharma et al.^26^ ^)^ described a high genetic diversity with low to high levels of genetic differentiation within populations of *E. granulosus* senso stricto (s.s.) from diverse geographical origins of all continents.

Previous studies on species that are transmitted in wildlife cycles, such as *E. vogeli* from Brazil,^16^ *E. multilocularis* from North America and Europe,^27^ and Tibetan *Echinococcus shiquicus* ^11^ reported higher nucleotide diversity (0.0044-0.0055) when compared to species transmitted to livestock animals, as *Echinococcus ortleppi* collected in five African countries and Brazil (0.0001-0.0008)^28^ and *E. granulosus* s.s. from China, Peru, Eastern Europe, and Italy (0.0002-0.0055).^11^ While the species with wild-life cycles seem to retain ancestral polymorphisms, those with domestic cycles show signs of genetic homogenisation due to animal transportation and introduction of small founder populations.^10^

To date, there is little information on the genetic structure of *E. vogeli*. The study of parasites in wild animals is laborious and sample collection in places with difficult access and with inadequate sanitary conditions lead to a great gap of knowledge concerning both the parasite and the disease. Our results differ from a previous study, in which a more comprehensive genetic structure was verified in populations from different Amazonian Brazilian states.^16^ ^)^ However, it is noteworthy that our sampling strategy included a more restricted geographical area. New sampling strategies are required to provide a better picture of the *E. vogeli* population dynamics in the Amazon.

Overall, our results showed that *E. vogeli* has undergone little migration in Acre and the presence of shared haplotypes among different populations seems to reflect the retention of ancestral polymorphisms. To the best of our knowledge, this is only the second study in the literature on the genetic structure and variability of *E. vogeli* populations. A better understanding of the dynamics, genetic structure and diversity of this species should help the diagnosis, epidemiology, and prevention of the disease, and contribute to taxonomical and evolutionary studies.
