# Supplementary material for: Genetic diversity of Echinococcus vogeli in the western Brazilian Amazon
Source: Mem Inst Oswaldo Cruz. 2019 Sep 26;114:e190149. doi: 10.1590/0074-02760190149 (PMC6764793; doi:10.1590/0074-02760190149)
Supplement: Supplementary file 1 [file 1678-8060-mioc-114-e190149-s.pdf]

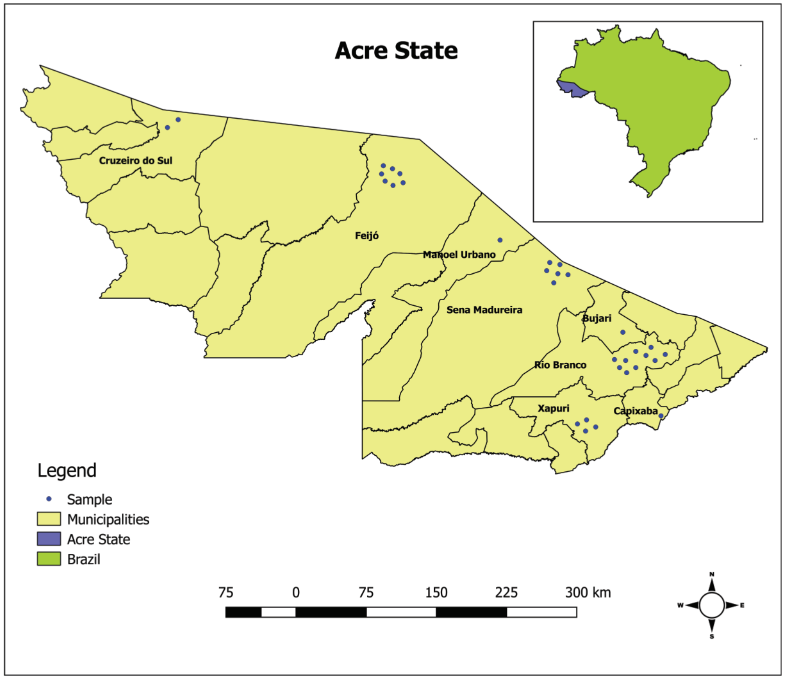

Map of Acre state, Brazil, showing the geographical origin of the collected *Echinococcus vogeli* samples.

TABLE

Characteristics of patients from the state of Acre, Brazil, who had surgically excised *Echinococcus vogeli* cysts

Sample ID	Sex	Age (surgery date)	Affected organ	Provenance
1	F	29	Liver	Sena Madureira
2	F	34	Liver	Capixaba
3	F	54	Liver	Rio Branco
4	M	51	Liver	Rio Branco
5	F	53	Liver	Cruzeiro do Sul
6	M	43	Liver	Sena Madureira
7	F	33	Liver	Rio Branco
8	F	31	Liver	Sena Madureira
9	M	40	Liver	Feijó
10	F	ND	Liver	Manuel Urbano
11	M	44	Liver	Xapuri
12	M	41	Liver	Feijó
13	M	38	Liver	Feijó
14	M	54	Liver	Sena Madureira
15	F	47	Liver	Xapuri
16	F	67	Liver	Feijó
17	F	58	Liver	Rio Branco
18	F	57	Liver	Rio Branco
19	F	63	Liver	Cruzeiro do Sul
20	F	73	Mesentery	Sena Madureira
21	F	67	Mesentery	Feijó
22	F	73	Liver	Rio Branco
23	F	40	Mesentery	Rio Branco
24	F	27	Liver	Bujari
25	F	53	Liver	Feijó
26	F	67	Liver	Rio Branco
27	M	53	Liver and mesentery	Rio Branco
28	M	55	Mesentery	Xapuri
29	M	63	Liver	Xapuri
30	F	42	Liver	Feijó
31	F	59	Liver	Rio Branco
32	F	27	Liver	Sena Madureira

F: female; M: male.

CC BY
